# Correspondence

**Published:** 1902-03

**Authors:** C. A. F. Lindorme


### CORRESPONDENCE.

*Editor Journal-Record of Medicine :*

Allow me to draw your attention to a gross neglect in the discussion of the Social Evil, which in your February number so deservedly has been given prominent space. Hitherto the criterion of usefulness of legal measures against it has been sought exclusively in the alternative of control by registration and independent practice, without all municipal attempt of hygiene to prevent pathologically serious consequences. This neglect is the examination of the *men*. If you run through the literature on the important, although little attractive topic, you find all the stress laid upon the possibility of feminine inspection. Why is that? Is not, under the registration system, the danger altogether with the men? If the women are under control, it is only the male *clièntele* which can carry cases in, and if these could be prevented, the house would be theoretically exempt. Now, the question is, whether inspection of the male moiety can be had. The possibilities have never been weighed here. But I think nothing can be more easily established: the bawd need only be placed under the corresponding obligation and responsibility, and they can use the girls themselves as inspectors. An instruction of the girls in that much of anatomy and pathological anatomy as to procure knowledge of the signs of infection would moreover be useful in other regards. It would impart to these psychologically lost cases of humanity some respect of their own personality, raise them somewhat above the mere classification of a tool, and this over and above a security which by the inspection of the women alone never can be attained.

Hence the ineffectiveness of the sequestering control is owing to the incompleteness of the measure. Without the masculine supplementing of the precaution the practical experience made does not tell. The insufficiency renders the police, even, indifferent in the matter.

But there is strenuous opposition against all municipal meddling with the regulation of Eros, the wild boy, not for reasons of health, but for reasons of *pruderie.* These by serious sociological students, should be discarded at once. Why, if we give room to such squeamishness, the fire brigade ought to go neither, if the house on fire be a brothel, and among the physicians the syphillogists ought to be a quite disreputable class of medical practitioners. Moreover these opposition tenderfoots do not know their own mind. They raise the argument that all check is taken from the vice if by the safety of its indulgence it is rendered so much more attractive. But at the same time, they raise the alarm, that under the sway of the worst hygienic conditions it is assuming dimensions as there never were before. There must be, therefore, another cause of the boom in that quarter, and this is without doubt the better financial position, generally. As long as dollars come sluggishly in the trades, it is certainly not the strumpets who notice latest that business is slackening up. Besides, if we must admit, that thousands of years of experience have taught us the impossibility to dislodge the affair from society, it is certainly wiser to treat the refractory element as a belligerant party, not as a revolting set; rather conclude, if regular peace cannot be had, a permanent armistice, instead of continuing a guerilla warfare which by its nature gives always the worst to the regulars.

If we go through the annals of legislation, on the field of sexual trespass, we notice that the lawgiver unexceptionally sided with the masculine moiety. This should be corrected. The opposite tactics is more useful. The interest in the women to escape disease is paramount. Prophylactic measures should, therefore, originate on their side. What can you do with a youngster who brags among his chums that he had a clap at an earlier age than anybody else, or an old tough that he had a bigger sore than his doctor ever saw before, and there is lots of this caliber. Women from the outset are not so terribly reckless, so, why not organisatorilly come to their rescue.

Let us cleanse society from this more than awful scourge of our civilization, the venereal diseases, be it even at the cost of the pretended diminition of the attractiveness of the vice; love-hunger, like food-hunger, is a self-limited affair. It is cured in its own surfeit. But the venereal diseases undermine the stamina of the human race. C. A. F. LINDORME, Ph.D., M.D.

